# HyperPUT: generating synthetic faulty programs to challenge bug-finding tools

**DOI:** 10.1007/s10664-023-10430-8

**Published:** 2024-01-15

**Authors:** Riccardo Felici, Laura Pozzi, Carlo A. Furia

**Affiliations:** 1 Computer Systems Institute, Università della Svizzera italiana (USI), Lugano, Switzerland; 2 Software Institute, Università della Svizzera italiana (USI), Lugano, Switzerland

**Keywords:** Program generation, Testing benchmarks, Synthetic bug injection, Testing frameworks, Fuzzing, Symbolic execution

## Abstract

As research in automatically detecting bugs grows and produces new techniques, having suitable collections of programs with known bugs becomes crucial to reliably and meaningfully compare the effectiveness of these techniques. Most of the existing approaches rely on *benchmarks* collecting manually curated real-world bugs, or synthetic bugs seeded into real-world programs. Using real-world programs entails that extending the existing benchmarks or creating new ones remains a complex time-consuming task. In this paper, we propose a complementary approach that automatically generates programs with seeded bugs. Our technique, called HyperPUT, builds C programs from a “seed” bug by incrementally applying program transformations (introducing programming constructs such as conditionals, loops, etc.) until a program of the desired size is generated. In our experimental evaluation, we demonstrate how HyperPUT can generate buggy programs that can challenge in different ways the capabilities of modern bug-finding tools, and some of whose characteristics are comparable to those of bugs in existing benchmarks. These results suggest that HyperPUT can be a useful tool to support further research in bug-finding techniques—in particular their empirical evaluation.

## Introduction

Research in detecting bugs automatically spans several decades, and has produced a wide array of diverse tools such as static analyzers, symbolic execution engines, and fuzzers—to mention just a few. In contrast to this long and successful history of developing bug-finding tools, there still is a somewhat limited agreement about how to rigorously evaluate and compare their bug-finding capabilities in realistic settings.

In the last few years, to address this conspicuous gap, we have seen several proposals of *ground-truth benchmarks*: curated collection of real programs including known bugs (Hazimeh et al. 2021) or seeded with synthetic bugs (Dolan-Gavitt et al. 2016; Roy et al. 2018), complete with detailed information about the bugs’ location, triggering inputs, and other fundamental characteristics. Ground-truth benchmarks have been instrumental in improving the rigor and thoroughness of bug-finding tools—especially those that generate test inputs using symbolic execution or fuzzing, which are the benchmarks’ usual primary focus. While the usefulness of ground-truth benchmarks is undeniable, extending a benchmark with additional bugs and programs—not to mention creating a new domain-specific benchmark from scratch—remains a complex and time-consuming endeavor.

In this paper, we explore a *complementary* approach to building ground-truth benchmarks, where we automatically generate from scratch programs with seeded bugs. The idea of constructing programs to be used as test inputs (PUTs: programs under test) has been successfully used for other purposes, such as to detect semantic compiler bugs that result in incorrect compilation (Yang et al. 2011).

Our technique, which we call HyperPUT, builds programs starting from a seed that consists of a simple block that fails when executed; this represents a seeded bug. Then, it repeatedly grows the program by adding features (branching, looping, and so on) that make it larger and more complex to test. HyperPUT is highly configurable: the user can choose aspects such as how many programs to generate, which syntactic features they should include, and the range of variability of their branching conditions. Clearly, there is no a priori guarantee that the synthetic PUTs generated by HyperPUT are representative of real-world bugs. However, a fully synthetic approach also has clear advantages over manually curated collections: since the whole process is automatic and customizable, producing new benchmarks collecting programs with specific characteristics is inexpensive. In addition, HyperPUT’s PUTs come with precise information about the bug location and any bug-triggering inputs. Thus, they can supplement the programs in curated ground-truth benchmarks to better evaluate the capabilities of bug-finding tools according to metrics such as number of discovered bugs and bug detection time, as well as to investigate which syntactic features of the faulty programs are more amenable to which bug-finding tools.

After discussing HyperPUT’s design and implementation in Section 3, in Section 4 we design some experiments where we generated hundreds of PUTs with bugs using HyperPUT, and we ran three popular, mature bug-finding tools—AFL++, CBMC, and KLEE—on these PUTs. Our goal is demonstrating that HyperPUT can generate bugs with diverse characteristics, which can challenge different capabilities of bug-finding tools and can usefully complement the programs in ground-truth benchmarks. To this end, we follow Roy et al. (2018)’s description of the features of “ecologically valid” bugs, and analyze whether HyperPUT can generate bugs that are fair, reproducible, deep, and rare, and that can exercise the different capabilities of common bug-finding techniques. The high-level summary of the experiments, which we detail in Section 5, confirms that HyperPUT is capable of generating “interesting” buggy programs that share some characteristics with those of benchmarks. Thus, HyperPUT can support flexible empirical analysis of the capabilities of the various bug-finding tools in a way that complements and extends what is possible using manually-curated benchmarks.

*Contributions* This paper makes the following contributions:

- HyperPUT, a configurable technique to automatically generate PUTs with certain characteristics and seeded bugs.
- An open-source implementation of the HyperPUT technique in a tool—also named HyperPUT.
- An experimental evaluation of HyperPUT that demonstrates its ability to generate bugs with characteristics comparable to “ecologically valid” ones (Roy et al. 2018), which exercise from different angles the capabilities of bug-finding tools.

The prototype implementation of HyperPUT is available in a public repository (HyperPUT 2022).

*Organization* The rest of the paper is organized as follows. Section 2 discusses the main related work in the development of benchmarks of bugs, as well as bug-finding techniques and tools. Section 3 describes the HyperPUT technique and its current implementation as a tool with the same name that generates programs in C. Section 4 introduces the paper’s research questions, and the experiments that we carried out to answer the questions. Section 5 presents the results of the experiments, and how they address the research questions. Finally, Sections 6 and 7 conclude with a discussion and summary of the paper’s contributions.

## Related Work

We discuss related work in two areas: benchmarks of bugs to evaluate bug-finding tools (Section 2.1), and the main techniques and tools to find bugs and vulnerabilities in programs (Section 2.2). Consistently with the paper’s main focus, we principally consider techniques and tools that work on programs written in the C programming language used for systems programming.

### Benchmarks of Bugs

Different applications of program analysis, including different approaches to test-case generation, use different benchmarks, consistent with the goals of the program analysis evaluated using the benchmark. Here, we focus on *extensible* benchmarks to evaluate the *bug-finding* capabilities of test-case generation frameworks (for brevity, testing framework).

**Table 1:** Classification of evaluation benchmarks according to whether they consist of organic or synthetic bugs within organic or synthetic programs (PUTs)

	Organic PUTs	Synthetic PUTs
Organic bugs	FuzzBench	
	MAGMA	
	CGC, Test-Comp, SV-Comp (datasets)	
Synthetic bugs	LAVA	CSmith
	Apocalypse	**HyperPUT**

Underlined systems support the automatic generation of new benchmarks by seeding bugs into existing programs

Table 1 shows a natural classification in terms of the origin of programs and their bugs, and displays the category several well-known benchmarks belong to. A *program* included in a benchmark can be *organic* or *synthetic*. The *bugs* of a benchmark’s PUTs can also be *organic* or *synthetic*.

*Organic programs* An *organic program* is one that was designed and implemented by human programmers, and hence reflects the characteristics of real-world programs (or at least a sample of them). For this reason, many existing benchmarks are based on organic PUTs. For example, the International Competition on Software Testing (Test-Comp) (Beyer 2021a) is a comparative evaluation of automatic tools for software test generation, which uses benchmarks consisting of C programs equipped with testing objectives (such as coverage, and bug finding). Similar benchmarks are used by the Competition on Software Verification (SV-Comp) (Beyer 2021). Another example is the CGC dataset, which collects about 300 small manually-written programs produced for the Darpa Cyber Grand Challenge (DARPA CGC 2018); for each bug in the programs, the CGC also includes a triggering input.

Google’s FuzzBench is an open benchmarking platform and service (Metzman et al. 2021) based on open source programs. FuzzBench has been useful both in the industrial and the academic fields—both to evaluate the capabilities of fuzzing frameworks and to identify their limitations and own bugs.

Organic benchmarks exist also for other programming languages, such as the DaCapo benchmarks (Blackburn et al. 2006) and Defects4J (Just et al. 2014) for the Java programming language.

*Synthetic programs* In contrast, a *synthetic program* is one that is generated automatically from a set of templates, rules, or heuristics.

CSmith (Yang et al. 2011) is a program generator mainly employed for validating compilers through differential testing (McKeeman 1998). It has been used to find several security problems in popular compiler frameworks (Marcozzi et al. 2019; Even-Mendoza et al. 2020), including GCC (Stallman 2023) and LLVM (Lattner and Adve 2004). Timotej and Cadar Kapus and Cadar (2017) applied a similar combination of grammar-based program generation and differential testing in order to find bugs in symbolic execution engines. While tools such as CSmith could be used to build benchmarks that challenge testing frameworks, they are most directly useful for *differential* testing, where the goal is comparing the behavior of different versions of a compiler. HyperPUT revisits some of the ideas behind tools like CSmith (in particular, grammar-based program generation) so that they are directly applicable to generate PUTs with seeded bugs. Differently from CSmith, HyperPUT can also produce a triggering input for each buggy program it generates, which serves as the ground truth to assess and compare the capabilities of different bug-finding tools.

*Organic bugs* An *organic bug* is one that occurred “in the wild”, and hence comes from a program’s actual development history. Just like organic programs, organic bugs have the clear advantage of being realistic. In fact, the majority of current systems for the evaluation of testing frameworks consist of organic PUTs and organic bugs. The MAGMA benchmark (Hazimeh et al. 2021) can extend the usability of such “fully organic” benchmarks by performing “forward-porting” of real bugs to recent version of the target PUT. This way, a historically relevant bug can still be reproduced (and tested for) in up-to-date setups. Still, applying MAGMA to new bugs and new PUTs requires substantial manual effort.

*Synthetic bugs* Seeding *synthetic* bugs into an existing program has become an increasingly popular approach to generate large benchmarks of bugs, thanks to its scalability compared to manual selection and curation. The Large-scale Automated Vulnerability Addition (LAVA) dataset (Dolan-Gavitt et al. 2016)—commonly used to compare fuzzing frameworks—consists of synthetic bugs seeded into existing programs. LAVA’s bug injection is based on the PANDA dynamic analysis platform (Dolan-Gavitt et al. 2015), built on top of the QEMU emulator (Bellard 2005). First, an analysis of the target program identifies dead, unused, and available (DUA) bytes of the input, which can be altered (“fuzzed”) without affecting the program’s behavior. Then, LAVA seeds vulnerabilities, such as buffer overflows or other kinds of inconsistent memory access, that are triggered when an execution accesses the DUA bytes.

Apocalypse (Roy et al. 2018) is a bug injection system similar to LAVA and based on synthetic bugs and symbolic execution. It generates and seeds into existing programs bugs with specific requirements (some of which we describe in Section 4 in relation to our experiments). Apocalypse was experimentally evaluated to show it can generate seeded bugs with characteristics comparable to organic ones. In Section 5, we will assess the PUTs generated by HyperPUT using several of the same metrics.

In order to work on real-world programs, LAVA and Apocalypse incur some limitations. First, one cannot seed bugs at arbitrary locations but only at those that have been reached in a previous execution. Second, since they rely on symbolic execution to discover triggering inputs for the seeded bugs, it may be practically hard to find such triggering inputs for bugs that are nested very deeply into the program’s control flow structure. Since HyperPUT builds PUTs with seeded bugs from scratch, it does not incur these limitations and can generate programs with arbitrarily complex nesting structure (as we demonstrate in Section 5.3).

Ferrer et al.’s work (Ferrer et al. 2011) is an example of fully synthetic benchmarks (consisting of synthetic bugs and synthetic PUTs) for the Java programming language. Their main goal is generating programs where every branch is reachable to serve as ground truth when evaluating the branch-coverage capabilities of testing frameworks.

Mutation testing is another approach based on injecting synthetic bugs in organic programs (Kontar et al. 2019; Kusano and Wang 2013). The original goal of mutation testing was to measure the bug-detection capabilities of a test suite: the more “mutants” (i.e., variants of program with injected bugs) trigger failures in the test suite, the more comprehensive the test suite is Pezzè M, Young et al. (2007). More recently, mutation testing ideas have been applied to different dynamic analysis techniques, such as fault localization (Papadakis et al. 2015; Bowes et al. 2016). As a bug-injection technique (Fraser and Zeller 2012), mutation testing suffers from the problem of *equivalent mutants*, which occur when a mutation does not alter a program’s behavior, and hence the mutant does not actually have a bug; a number of approaches have tried to address this problem (Papadakis et al. 2015; Yao et al. 2014; Schuler and Zeller 2013).

It is also interesting to consider which metrics are supported by the benchmarks. The most common ones are number of detected bugs, detection time, and maximum code coverage achieved during testing; these are easily applicable to all benchmarks. In addition, one may want to relate the syntactic features of the buggy programs to the capabilities of the bug-finding tools; HyperPUT’s approach supports this kind of experiments, since it can generate batches programs with similar characteristics (e.g., nesting structure or kinds of statements).

### Bug-Finding Tools

A detailed discussion of the main techniques used to find bugs in programs is beyond the scope of the present paper; we refer the interested readers to surveys (Candea and Godefroid 2019; Cadar and Sen 2013; Baldoni et al. 2018) and textbooks (Ammann and Offutt 2007; Pezzè M, Young et al. 2007). In this section, we briefly describe the bug-finding techniques and tools that feature in our evaluation of HyperPUT—which are also widely used outside of research.

*Fuzzing* Fuzz testing (or *fuzzing*) encompasses a broad spectrum of dynamic techniques to generate program inputs (Manès et al. 2021; Klees et al. 2018). It is widely used to find bugs in software; Google, for instance, found thousands of security-related bugs in their software using fuzzing (Babic et al. 2019). The key idea of fuzzing is to *randomly mutate* a known valid program input (the “seed”) to generate new inputs that may cause the program to crash or expose other kinds of vulnerabilities. Fuzzers differ according to the kind of strategies they use to randomly mutate program inputs. In particular, black-box fuzzers do not have access to the target program’s control flow, and hence can only generate new inputs independently of the program’s structure. In contrast, white-box fuzzers can take the program’s control flow into account in order to generate new inputs that exercise specific portions of the program.

American Fuzzy Lop (AFL) is one of the most popular fuzzing frameworks for C programs. It is a gray-box coverage-based fuzzer, which means that some of its fuzzing strategies are driven by coverage information about the analyzed program. Originally developed by Zalewsky (Zalewski et al. 2016), different extensions of AFL —such as RED-QUEEN (Aschermann et al. 2019), AFLFast (Böhme et al. 2016) and AFL++ (Fioraldi et al. 2020)— have been introduced more recently and remain widely used.

*Symbolic execution* As the name suggests, symbolic execution executes a program with *symbolic* inputs, which are placeholders for every possible valid inputs (Cadar and Sen 2013; Baldoni et al. 2018). As it enumerates different execution paths, symbolic execution builds *path constraints*, which are logic formula that encode each path’s feasibility. Then, a constraint solver such as Z3 (Moura and Bjørner 2008) determines which abstract paths are feasible, and generates matching concrete inputs.

Most modern implementations of symbolic execution perform *dynamic symbolic execution* (also called “concolic” execution), which combines symbolic and concrete state in order to overcome some limitations of symbolic execution (such as its scalability and applicability to realistic programs) (Bornholt and Torlak 2018). EXE (Cadar et al. 2008) and DART (Godefroid et al. 2005) pioneered the idea of dynamic symbolic execution. More recently, other tools perfecting and extending this technique include KLEE (Cadar et al. 2008), SAGE (Godefroid et al. 2012), S2e (Chipounov et al. 2012), and Angr (Shoshitaishvili et al. 2016). KLEE is one of the most widely used dynamic symbolic execution engines for C programs. It is implemented on top of LLVM (Lattner and Adve 2004), and has been successfully employed to find several bugs in production software, such as the MINIX (Tanenbaum et al. 2010) and BUSYBOX (Busybox 2023) tools.

Driller is a vulnerability discovery tool that combines symbolic execution and fuzzing (Stephens et al. 2016). When the latter fails to make progress, it uses the former to continue the exploration of new execution paths. This approach is effective to improve code coverage, and to test features such as cryptographic hash functions and random number generators, which are notoriously difficult for approaches that are exclusively based on constraint solving. The T-Fuzz fuzzer (Peng et al. 2018) applies program transformations in order to remove the conditions guarding some code blocks that are hard to reach. If a crash occurs in these code blocks, it then checks a posteriori whether the locations are actually reachable in the original program.

*Model checking* In a nutshell, model checking is a verification technique for finite-state models, which can exhaustively check properties expressed in temporal logic (including reachability properties, which can be expressed as assertions in the code) or find counterexamples when the properties do not hold in general (Clarke et al. 1986).

Since real-world programs are not finite state, one needs to introduce some kind of finite-state abstraction in order to be able to apply model checking to them. A natural way of doing so is by *bounding* the program state to be within a finite (but possibly very large) range. Then, model checking such a bounded abstraction is not equivalent to verifying the original program, but can still be a very effective way of thoroughly *testing* the program and finding bugs. In this paper, we experiment with the popular CBMC (Clarke et al. 2004) bounded model checker for C programs.

An alternative classification for testing framework benchmarks is based on the employed evaluation criteria. The most common ones are the detection time for a particular bug, the number of detected bugs in the benchmark and the code coverage testing frameworks achieve during program execution, measured in terms of number of lines or branches visited in the PUT control flow graph. Every benchmark described in this section supports the first two mentioned criteria, while coverage measurements can be easily incorporated at compilation time. HyperPUT, in addition, can also evaluate testing frameworks depending on the structure of the produced PUT, as described in Section 3.

## Methodology and Implementation

HyperPUT builds arbitrarily complex PUTs by recursively applying parametric transformations of different kinds to an initial simple program.

**Table 2:** HyperPUT’s transformations and the corresponding generated code

Transformation	Category	Code
	C	
	C	
	L	
	W	
	W	

In a transformation, lowercase letters denote parameters and uppercase letters denote holes. Each transformation belongs to one of three main categories: comparisons (C), loops (L), and widgets (W)

### Transformations

A *transformation* consists of a program *template* with (typed) parameters and holes. When we *apply* a transformation, we choose concrete values for its parameters and holes. A parameter can be replaced with any constant or variable of suitable type. A hole is replaced by another snippet of code, which can be given explicitly or as the result of nesting another transformation. Table 2 lists the transformations HyperPUT currently supports, together with the code they correspond to. There are five main kinds of transformations:

**IC**: **(integer comparison)** introduces a conditional that checks whether the two integer parameters $v_1, v_2$ are equal.
**SC**: **(string comparison)** introduces a conditional that checks whether the two string parameters $s_1, s_2$ are equal.
**FL**: **(for loop)** introduces a loop that iterates *e* times (where *e* is the transformation’s integer parameter), and then executes code *B*.
**PC**: **(palindrome check)** introduces a loop that checks whether the string parameter *s* is a palindrome of length at least *n*; if it is, it executes code *B*.
**CC**: **(character counting)** introduces a loop that counts the number of occurrences of character parameter *c* in string parameter *s*; if the count equals the integer parameter *n*, it executes code *T*; if not, it executes code *E*.

Let’s present a few more details about transformation IC, as an example to illustrate how transformations work. Transformation IC consists of two parameters $v_1$ and $v_2$ and two holes *T* and *E*. The parameters denote two integer values or variables. Then, the transformation introduces a conditional  that checks whether $v_1$ and $v_2$ have the same value. If they have, *T* executes; otherwise, *E* executes.

#### Transformation Categories

To present the rationale behind this selection of transformations, it is useful to classify them into three broad categories: transformations IC and SC are pure *conditionals*; transformation FL consists of a *loop*; and transformations PC and CC are more complex combinations of conditions and iterations that capture *widgets* (that is, simple parameterized algorithms).

We selected these transformations to demonstrate how HyperPUT can generate, from a small number of basic programming elements, a large number of PUTs that can effectively challenge different testing frameworks, and some of whose features are comparable to those of curated bug collections. To this end, we introduced elementary conditional transformations that are based on string and integer comparisons; these are frequently sources of serious bugs and vulnerabilities in real-world C programs (Younan et al. 2012), and can result in PUTs that are challenging to analyze for techniques such as black-box and gray-box fuzzing (Stephens et al. 2016). To make the PUTs more diverse and to add layers of complexity, we also introduced loops and widgets (which algorithmically combine loops and conditionals): loops complicate the control flow of the generated PUTs (increasing measures such as cyclomatic complexity, as discussed in Section 4.6.1), and widgets introduce complex feasibility constraints, which can challenge even testing frameworks based on constraint solving (such as KLEE).

Despite these considerations, the currently supported transformations are not meant to capture all—or even a large part of—the variety and complexity of real-world C programs. This paper’s goals are simply to demonstrate the potential usefulness of HyperPUT. In future work, users may add or change HyperPUT’s transformations according to their specific goals and needs.

### Transformation Sequences

More complex PUTs combine several transformations by nesting one inside another. When we specify a *sequence* of transformations, we can give a concrete value to any transformation *parameter* or use a fresh identifier. In the latter case, HyperPUT will instantiate the parameter with a suitable random value (usually within a range)—for every PUT generated from the transformation sequence. For example, the expression , where $\beta$ is a fresh identifier, denotes a conditional that checks whether the first command-line argument  , when interpreted a s an integer, is equal to a random integer value; if it is, the program fails  , otherwise, it exits normally  .

**Fig. 1:**
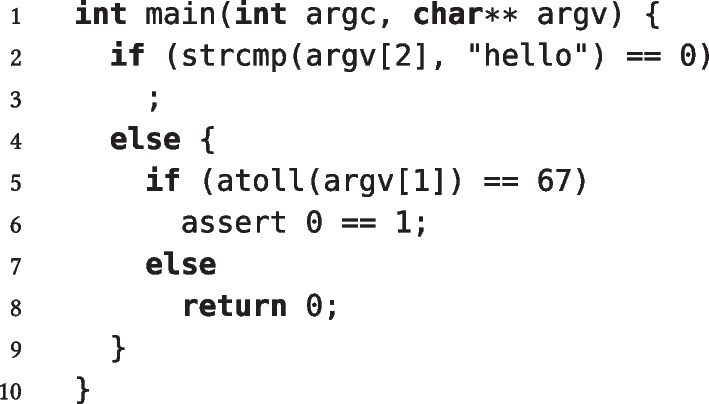
Specification of a PUT that combines transformations SC and IC as in (1)

We can also use fresh identifiers, instead of concrete code snippets, for *holes*, to denote that the next transformation in the sequence will instantiate the hole. In other words, this is just a notational shorthand that helps readability by avoiding nesting transformations explicitly. For example, the sequence of two transformations1nests an integer comparison inside the else branch of a string comparison, and thus it is equivalent to the explicitly nested expressionand determines the PUT in Fig. 1.

Figure 1 also shows that HyperPUT inserts the code generated by applying a sequence of transformations into a template main function, so that the PUT is a complete program. HyperPUT also automatically generates boilerplate code—such as library includes, and checks that the required command-line arguments are indeed present— that makes PUTs syntactically correct programs. For simplicity, Fig. 1 and all other PUTs shown in the paper omit this boilerplate code.

#### Reaching Inputs

The structure of every transformation suggests which values of the transformation’s parameters determine an execution of the resulting PUT that reaches code in any of the transformation’s holes. For example, hole *T* in transformation IC executes for any $v_1 = v_2$; hole *B* in transformation FL always executes; hole *T* in transformation CC executes if *s* includes *n* occurrences of characters *c*; and so on. In Fig. 1’s example, there are two variables  and  , and three leaf holes at lines 3, 6, and 8; hence, the inputs , , and  respectively reach each of the leaves.

For each transformation in Table 2, HyperPUT is equipped with an *input-generation* function that returns concrete values for the transformation’s typed parameters that reach any of the holes in the transformation’s code. For transformations IC and SC, the input-generation function simply draws a random integer *v* (IC) or string *s* (SC); for transformation FL, the input-generation function draws a random positive integer *e*; for transformation PC, the input-generation function constructs a palindrome string *s* of length *n* by randomly constructing a string *t* of length *n*/2, and then by concatenating *t* and its reversal to construct *s*; for transformation CC, the input-generation function constructs a random string *s* with *n* random occurrences of character *c*.

Using input-generation functions, HyperPUT can—under certain conditions—construct a program input that reaches the bug location. Precisely, consider a transformation sequence such that:

*i)* In every occurrence of transformation IC, exactly one parameter $v_1$ or $v_2$ is instantiated with  , for some positive integer *k*; *ii)* In every occurrence of transformation SC, exactly one parameter $s_1$ or $s_2$ is instantiated with  , for some positive integer *k*; *iii)* In every occurrence of transformations PC and CC, parameter *s* is instantiated with  , for some positive integer *k*; *iv)* In the whole transformation sequence, any  occurs at most once.

Under these restrictions, HyperPUT simply collects the values returned by each transformation’s input-generation function along the path that reaches the seeded bug, and concatenates them to instantiate the generated PUT’s input arguments  , which HyperPUT returns as the bug-triggering input. Consider Fig. 1’s example again; the corresponding transformation sequence (1) satisfies the above constraints. Hence, HyperPUT uses  as the triggering input for  and  (a random string different from  ) as the triggering input for  ; altogether,  is the input reaching the assertion failure at line 6.

As we discuss in Section 4.2, all the transformation sequences used in our experiments satisfy these constraints, so that HyperPUT can generate suitable triggering inputs for every PUT. In some cases, a PUT admits multiple triggering inputs; by default HyperPUT returns only one of them, randomly, but it can also produce additional ones. Finally, if we supply a transformation sequence that does not satisfy the above constraints (which can be determined with a simple syntactic check), HyperPUT may fail to generate any triggering input, although it may still generate valid PUTs with a “best effort” approach.

### Implementation Details

We implemented the HyperPUT technique in a tool with the same name. The tool is implemented in a combination of C (for the core program-generation functionalities), Python (front end and connection of the various modules), and Bash scripts (to run batches of experiments).

The user input to HyperPUT consists of a sequence of transformations specified as described in Section 3.2, and a number of PUTs to be generated. HyperPUT’s *front end* processes this input and passes the information to the *generator engine*, which takes care of generating PUTs by applying the transformation sequences, embedding the resulting code into a main function to build a complete program, and also recording a reaching input for every generated PUT.

*Extensibility* HyperPUT is extensible with new transformations. However, as we demonstrate in Section 5, the current selection of transformations is already sufficient to generate a large number of “interesting” PUTs, which can challenge different test-case generators and share some characteristics with the programs in widely used test-case generation benchmarks.

In principle, HyperPUT’s pipeline could also generate PUTs in programming languages other than C. To this end, one should extend it with transformations that generate valid snippets of code in other programming languages.

**Table 3:** Configurations of the testing tools used in the experiments

ID	Framework	Configurations
*A*	AFL++	afl-clang-fast with options CMPLOG (Cmplog instrumentation 2023), LAF (Circumventing fuzzing roadblocks with compiler transformations 2016), MOpt (Lyu et al. 2019)
		afl-clang-fast with default options
*C*	CBMC	automated bounded loop unwinding
		loop unwinding with bound 10
*K*	KLEE	symbolic arguments, random state search, LLVM optimization
		symbolic arguments, default options

Each row specifies two configurations for a testing tool in terms of the used options

## Experimental Design

The experimental evaluation of HyperPUT addresses the following research questions:  

**RQ1:** Can HyperPUT generate bugs that are *fair*?
**RQ2:** Are the bugs generated by HyperPUT *reproducible*?
**RQ3:** Can HyperPUT generate bugs that are *deep* and *rare*?
**RQ4:** Can HyperPUT generate diverse programs that exercise different *capabilities* of bug-finding techniques?

  This section describes the experiments we designed to answer these research questions. Our experimental design is after Roy et al. (2018)’s, modified to suit our goal of evaluating the characteristics of HyperPUT’s synthetic PUTs.

### Testing Frameworks

To assess the characteristics of the bugs generated by HyperPUT, we ran several testing frameworks on the generated PUTs and determined which bugs each framework could uncover.

We used testing frameworks implementing different bug-finding techniques for C programs:

- AFL++ (Fioraldi et al. 2020) is a popular grey-box fuzzer, which combines random generation of input and coverage metrics. It extends the original AFL (Zalewski et al. 2016) with several research improvements.
- CBMC (Clarke et al. 2004) is a bounded model checker for C/C++ programs. Bounded model-checking exhaustively explores a program’s state-space up to a finite size bound, checking for the violation of basic correctness properties (such as memory safety) and assertions within this explored space.
- KLEE (Cadar et al. 2008) is a state of the art dynamic-symbolic execution engine. Dynamic-symbolic execution is a white-box testing technique, which uses constraint solving to generate inputs that lead to exploring new paths in the PUT.

**Table 4:** List of the batches of PUTs used in HyperPUT’s experimental evaluation to answer RQ1, RQ2, and RQ3

Batch	*n*	#puts	Inputs used as parameters $v_1, s_1, s$
$B_1$	1	10	
$B_2$	2	45	
$B_{10}$	2–10	200	
$B_{100}$	100	100	
$B_{1000}$	1000	100	

For each batch, the table lists the number *n* of *transformations* used to generate each PUT in the batch, the number #puts of different PUTs in the batch, and the command-line input arguments used as parameters in the transformations

**Table 5:** Range of values, between a minimum and a maximum value, for the parameters of the transformations in Table 2 used in the experiments

Transformation	Parameter	Min	Max
IC	$v_2$		
SC	$s_2$		
FL	*e*		
PC	*n*		
CC	*n*		

These tools offer numerous configuration options; Table 3 lists the configurations that we used in the experiments. We deploy each tool in two configurations: we first execute it with its first configuration; if it fails to find a bug before the timeout expires, we execute it again on the same PUT with its second configuration (using any remaining time). For brevity, henceforth we use the expression “we run *X* on a program *P*” to mean “we run the testing framework *X* using sequentially the two configurations in Table 3 on *P*”.

### Experimental Subjects

We generate PUTs in batches, where each batch runs HyperPUT with a sequence of $n \ge 1$ transformations:2and a matching sequence of actual parameters $p_{1,1}, p_{1,2}, \ldots , p_{n,1}, p_{n,2}, \ldots$. Each transformation $T_k$ in (2) is one of IC, SC, FL, PC, and CC listed in Table 2. In the experiments, we always nest into the “then” hole *T* of conditional transformations IC and SC; therefore, all “else” holes *E* are simply filled with a “skip” snippet that does nothing. Snippet  indicates code that triggers a crashing bug when executed (i.e., an assertion failure  . In our experiments, we always add the snippet  in the innermost transformation $\textsf {T}_n$.

Each actual parameter $p_j$ is either a random constant of the appropriate type (chosen within a limited range) or *i)*  (for , as shown in Table 4) for parameters of type  ; *ii)*  (for ) for parameters of integer type (  ). More precisely, parameters $v_1$ in transformation IC, $s_1$ in transformation SC, and *s* in transformations PC and CC are always instantiated with a command-line argument; all other parameters are chosen as random constants within a small range. Table 5 shows the actual ranges for the randomly chosen parameters in each transformation in the batches that we used in the experiments. For example, every instance of IC uses an integer between 0 and 255 as its second parameter $v_2$.

Finally, in each PUT that consists of *n* transformations, each command-line argument  , for $1 \le k \le n$, is used exactly once.

We introduce these restrictions on the choice of parameters so as to generate PUTs of homogeneous characteristics, where the number and kinds of transformations used to generate them are the primary determinant of their complexity. These constraints also ensure that, in every generated PUT, *i)* there is exactly one bug; *ii)* there is (at least one) program input that triggers the bug.

As we discussed in Section 3.2.1, under these conditions HyperPUT can automatically construct a reaching input for the unique bug’s location, which is thus also a triggering input that ensures that the bug is executable.

#### Batches

For the experiments with HyperPUT to answer RQ1, RQ2, and RQ3, we generated a total of 455 PUTs in 5 batches. Table 4 outlines the characteristics of each batch.  

**Batch**: $B_1$ includes 10 PUTs, each consisting of a single transformation.
**Batch**: $B_2$ includes 45 PUTs, each consisting of two different transformations.
**Batch**: $B_{10}$ includes 200 PUTs, each consisting of between 2 and 10 transformations (possibly with repetitions), with the transformations and the actual length chosen randomly. More precisely, this batch includes: *i)*1 PUT consisting of 2 transformations; *ii)* PUTs consisting of 3 transformations; *iii)* 9 PUTs consisting of 4 transformations; *iv)* 41 PUTs consisting of 5 transformations; *v)* 44 PUTs consisting of 6 transformations; *vi)* 43 PUTs consisting of 7 transformations; *vii)* 29 PUTs consisting of 8 transformations; *viii)* 20 PUTs consisting of 9 transformations; *ix)* 9 PUTs consisting of 10 transformations. The rationale for selecting the PUTs in batch $B_{10}$ is as follows. The bulk of the PUTs are of intermediate sizes (5, 6, and 7 transformations), which hit a sweet spot in effectively exercising the various testing frameworks: they are neither trivial nor excessively difficult to analyze. Then, a decent number of larger PUTs (8, 9, and 10 transformations) demonstrate the limits of the various testing frameworks. The smaller PUTs (2, 3, and 4 transformations) are not particularly discriminating; we include a few for completeness’s sake, but adding more would not significantly change our experimental results.^1^
**Batch**: $B_{100}$ includes 100 PUTs, each consisting of exactly 100 transformations (possibly with repetitions) chosen randomly.
**Batch**: $B_{1000}$ includes 100 PUTs, each consisting of exactly 1000 transformations (possibly with repetitions) chosen randomly.

**Fig. 2:**
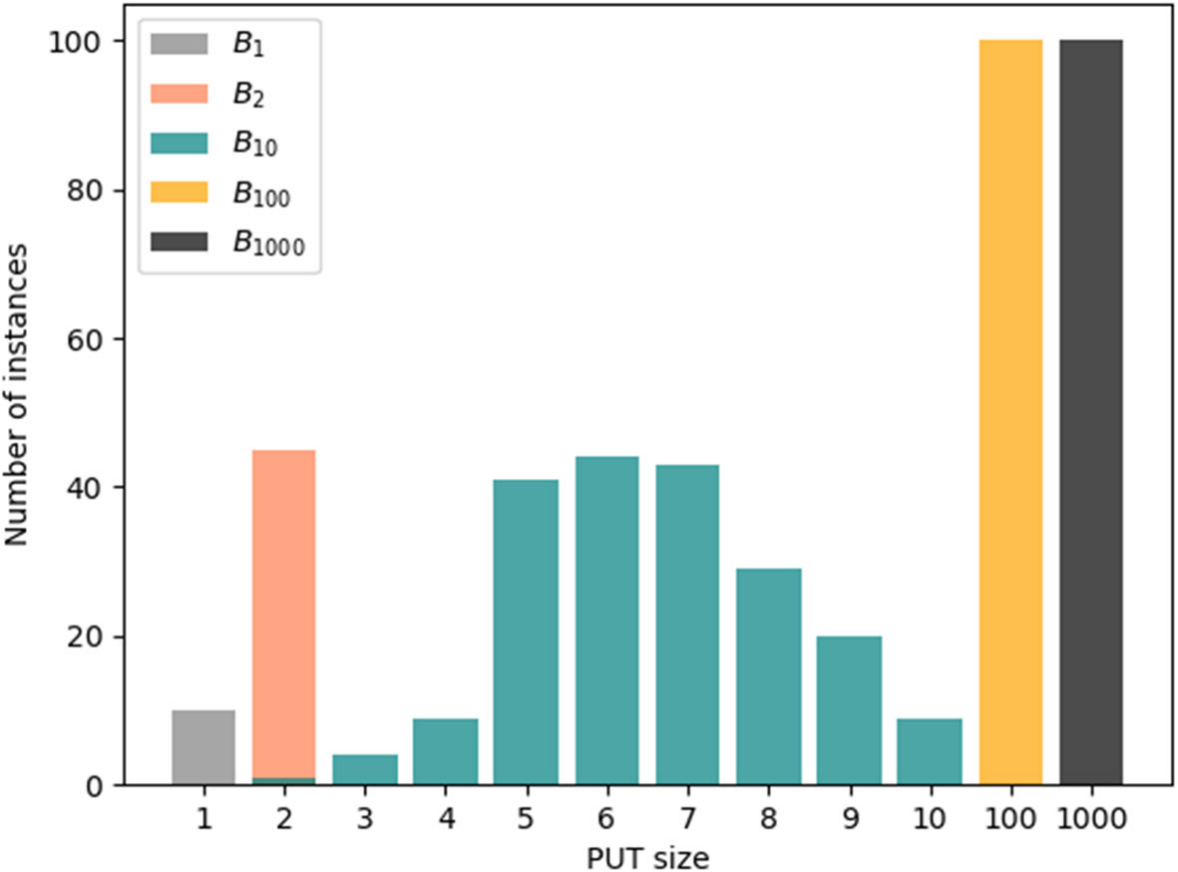
Distribution of size (in number of transformations) of the PUTs used in the experimental evaluation

Henceforth, *B* denotes the union of all batches $B_1 \cup B_2 \cup B_{10} \cup B_{100} \cup B_{1000}$. Figure 2 overviews the distribution of all PUTs in *B*.

For the experiments with HyperPUT to answer RQ4, we generated another 60 PUTs in 6 batches $B_{\textsf {IC}}, B_{\textsf {SC}}, B_{\textsf {FL}}, B_{\textsf {PC}}, B_{\textsf {CC}}, B_{\star }$. For each transformation *T* among IC, SC, FL, PC, and CC, batch $B_{T}$ consists of 10 PUTs $P_T^1, \ldots , P_T^{10}$. Each PUT $P_T^m$ corresponds to the sequence of transformations3with *m* transformations, all equal to *T*. In other words, $B_{T}$ consists of increasingly long sequences of the same transformation *T* repeated multiple times. Similarly, batch $B_\star$ consists of 10 PUTs $P_\star ^1, \ldots , P_\star ^{10}$; each PUT $P_\star ^m$ corresponds to the sequence of transformations (2), with $n=m$ transformations, each transformation randomly chosen (possibly with repetitions) among IC, SC, FL, PC, and CC.

#### Seeded Bugs

In principle, HyperPUT’s seeded bugs can be any piece of code, matching any kind of error; in our experimental evaluation, however, all seeded bugs are simply assertion failures. This is consistent with how the capabilities of testing frameworks are commonly evaluated on (curated) collections of bugs: an experiment challenges a testing framework to generate a program input that *reaches* an *error location* in the PUT. Since our experimental evaluation aims at demonstrating whether HyperPUT can generate bugs with *some* characteristics comparable to those in manually-curated benchmarks, using assertions (which are equivalent to reachability properties) as seeded bugs is a reasonable choice.

Besides being commonly used as targets of fuzzing techniques (Candea and Godefroid 2019; Malik and Pastore 2023; Payer 2019), assertions can model a broad range of *safety* properties (Lamport 1977). These include memory-related errors (e.g., violating  corresponds to a null pointer dereferencing error), as well as (partial) correctness errors (e.g., violating  corresponds to a postcondition error), but exclude liveness properties such as termination.^2^

### Experimental Setup

We ran all experiments on an Intel® Core^TM^ i5 machine with 2 cores and 8 GB of RAM running Ubuntu 18.04 Bionic, LLVM 6.0.1, AFL++ 2.68c, CBMC 5.10, and KLEE 2.1.

Every PUT generated by HyperPUT accepts command-line arguments as input for its  function. This is the only input that a testing tool controls when testing a PUT. For example, when running KLEE, the command line argument array  is instrumented with  , and the rest of the PUT is unmodified.

Each experiment runs one of the tools in Table 3 on a PUT with a timeout of 1 hour. The outcome is success if the testing framework successfully generates command-line inputs that trigger the  injected bug in the PUT. To accommodate fluctuations due to the operating system’s nondeterministic scheduling, as well as in possible randomization used by the testing frameworks, we repeat each experiment four to ten times, and report the average wall-clock running time as the experiment’s duration. The outcome is success if at least one of the repeated runs is successful (i.e., it triggers the bug).

### RQ1: Fairness

A collection of bugs is *fair* if state-of-the-art bug detection techniques, especially those that are widely used in practice, can discover the bugs with reasonable effort; and if it is not strongly biased in favor or against any one detection technique. For a PUT-generation system like HyperPUT, *fairness* means that it should be capable of generating bugs with a broad spectrum of “detection hardness”—from simple to very challenging to discover.

To demonstrate *fairness*, we ran each of the tools AFL++, CBMC, and KLEE on all PUTs in *B*. We then analyzed which tools were successful in triggering the bugs in the PUTs within the timeout.

In particular, the percentage of bugs that is detected by at least one of the tools is a fundamental measure of fairness. As a rule of thumb (Roy et al. 2018), this percentage should be definitely above 50%, meaning that the majority of bugs are *discoverable* given the (combined) capabilities of the testing frameworks. Conversely, this percentage should not be too close to 100%; if it were, it would mean that the bugs are not sufficiently *elusive*, failing to fully stress the testing frameworks’ capabilities.

### RQ2: Reproducibility

A bug is *reproducible* if there is a known input that consistently triggers the bug. For a PUT-generation system like HyperPUT, *reproducibility* also entails that the PUTs compile without errors and do not rely on any undefined behavior of the C language.

As explained in Section 3.2, all PUTs generated by HyperPUT in our experiments have a unique bug and should come with an input that triggers it.

To assess *reproducibility*, we ran each PUT generated in the experiments with the triggering input, and checked whether the bug was triggered as expected.

HyperPUT generates PUTs that should be syntactically and semantically correct. To confirm this, we compiled each PUT generated in the experiments using both GCC (with options  and  ) and LLVM (with options  and  ), and checked that: *i)* both compilations succeeded without errors; and *ii)* both compiled versions behaved in the same way—namely, they fail when executed with the triggering input. To detect the potential presence of undefined behavior, we also checked every generated PUT using LLVM’s Undefined Behavior Sanitizer (Undefined behavior sanitizer (ubsan) 2023), a compiler instrumentation that can detect several instances of undefined behavior.

### RQ3: Depth and Rarity

Depth and rarity are two different ways of assessing the “hardness” of a bug for bug-detection techniques.

#### Depth

A bug is *deep* if triggering it requires to follow a long sequence of statements and branches. For a PUT-generation system like HyperPUT, bug *depth* depends on the structure and complexity of the PUTs themselves. To determine whether HyperPUT’s bugs are *deep*, we measured the following on every PUT in batch *B* generated in the experiments:

- The cyclomatic complexity of the PUT.^3^ Cyclomatic complexity (McCabe 1976) is a static measure of complexity of a program’s branching structure, which counts the number of distinct simple execution paths a program has. Cyclomatic complexity is used as a fundamental measure of control-flow (and cognitive) complexity (SWE-220 2023), which negatively correlates with software qualities such as testing coverage (Kochhar et al. 2014). Procedures with cyclomatic complexity higher than 10 usually correspond to programs that are challenging to analyze (Lanza and Marinescu 2006). In order to assess the complexity of HyperPUT’s PUTs compared to that of programs in other benchmarks, we compare the cyclomatic complexity of PUTs in *B* to that of programs in CGC (DARPA CGC 2018) and LAVA-1 (Dolan-Gavitt et al. 2016). Note that the PUTs generated by HyperPUT consist of a single main function, but programs in other benchmarks usually consist of several different functions; thus, we measure the cyclomatic complexity of each function in the programs in isolation, and report statistics about their distribution in each benchmark. We only measure the cyclomatic complexity of functions in the actual PUTs, not in any external library that is used by the PUTs.
- The length (in number of instructions executed at runtime) of the execution path that goes from the PUT’s entry to the bug-triggering statement, when the PUT is executed with a triggering input.^4^ Path length is a dynamic measure of how deep a bug is within a path that triggers it. Similarly to cyclomatic complexity, we compare the path length of bugs in *B* to that of bugs in benchmark LAVA-1. Path length complements cyclomatic complexity as a measure of depth:
  *i)*: path length is a *dynamic* measure, whereas cyclomatic complexity is static;
  *ii)*: path length directly measures the depth of a *bug*, whereas cyclomatic complexity measures the complexity of the PUT that contains a bug.

#### Rarity

A bug is *rare* if it is only triggered by a small fraction of all possible program inputs. To determine whether HyperPUT is capable of producing PUTs with *rare* bugs, we followed the same protocol of Roy et al. (2018): we ran KLEE on each buggy PUT with a timeout of 1 hour and measured the following.

- The number *f* of test cases generated by KLEE before first triggering the bug.
- The number *t* of test cases, among those generated within the timeout, that trigger the bug.

These measures give an idea of how sparse the bug-triggering inputs are in the space of all inputs that are generated by a systematic strategy.

In order to be able to compare HyperPUT’s measures of rarity with those of other benchmarks’, we only considered PUTs in batch $B_{\ge 6}$ for this experiment. Batch $B_{\ge 6}$ consists of the 72 PUTs in $B_{10}$ with 6, 7, 8, 9, or 10 transformations that KLEE can discover within the 1-hour timeout. We exclude PUTs whose bugs KLEE cannot uncover, as the measures *f* and *t* are undefined in these cases.We also exclude PUTs that are much smaller (e.g., $B_{2}$) and much larger (e.g., $B_{100}$). PUTs larger than ten transformations are exceedingly unlikely to be detectable by KLEE, and hence they are not relevant to assess rarity. As for the smaller PUTs, we deliberately exclude them as they are not representative of rare bugs. This is consistent with how we addressed every question in this empirical evaluation: HyperPUT is a highly configurable tool, which can produce PUTs with different characteristics. In answering RQ3, our aim is demonstrate how one can configure HyperPUT to produce PUTs with rare bugs. If rarity is not a desired property to the user, HyperPUT may be configured differently to produce PUTs with other characteristics.

We compare these metrics of rarity for HyperPUT to those reported by Roy et al. (2018) for 41 manually seeded bugs in the TCAS benchmark (Do et al. 2005), as well as 82 synthetic bugs seeded using their Apocalypse system in the same TCAS programs. More precisely, (Roy et al. 2018, Table 4) reports the number of all bug-triggering tests generated by KLEE within 1 hour, which corresponds to measure *t*. In addition, (Roy et al. 2018, Fig. 5) plots the number of tests generated by KLEE before hitting a first bug, which corresponds to measure *f*. We directly compare these to the same measures on HyperPUT’s PUTs, without repeating (Roy et al. 2018)’s experiments. We only use KLEE to investigate rarity both because it is a standard choice for this kind of assessment (Roy et al. 2018), and because its systematic exploration of program paths provides a more robust measure than others (such as testing time) that are strongly affected by the sheer size and complexity of the PUT as a whole—as opposed to its bugs’ specifically.

### RQ4: Capabilities

To further demonstrate the flexibility of HyperPUT’s generation, we look more closely at how different bug-finding tools perform on different batches of PUTs generated by HyperPUT. Which PUTs are easier or harder to analyze suggests which capabilities of the bug-finding tools are more or less effective to analyze programs with certain features.

**Table 6:** For each combination of tools, for each batch of PUTs used in the experiments, the percentage % and the absolute number # of PUTs in the batch whose unique bugs were triggered by the tests generated by those tools

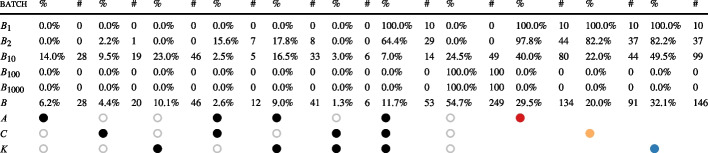	

The leftmost columns report bugs triggered *exclusively* by each tool combination (those marked by  in each column); the rightmost columns report bugs triggered *non*-exclusively by each tool (those marked by  for AFL++,  for CBMC, and  for KLEE). For example, the leftmost column indicates that tool *A* managed to find bugs in 28 PUTs in batch *B* (6.2% of all PUTs in *B*), which no other tool could find; the rightmost column indicates that tool *K* managed to find bugs in a total of 146 PUTs in batch *B* (32.1% of all PUTs in *B*)

We ran each of the tools AFL++, CBMC, and KLEE on the PUTs in $B_{\textsf {IC}}$, $B_{\textsf {SC}}$, $B_{\textsf {FL}}$, $B_{\textsf {PC}}$, $B_{\textsf {CC}}$, and $B_{\star }$. Since these batches include multiple repetitions of the same transformation, they demonstrate the generation of PUTs with homogeneous characteristics. By observing how each tool’s bug-finding capabilities change in different batches, and within each batch as the same transformation is repeated multiple times, we can outline each tool’s strengths and weaknesses in comparison with the other tools’ and link them to the characteristics of the transformations.

## Experimental Results

### RQ1: Fairness

Table 6 reports, for each batch of PUTs in Table 4, which testing tools were able to generate inputs triggering the PUTs’ unique bugs in our experiments. Row *B* corresponds to all PUTs used in these experiments. At least one of the tools *A*, *C*, and *K* managed to detect bugs in 80.8% of all PUTs with less than 100 transformations.^5^ The distribution is not strongly biased in favor of any tool—even though *K* was noticeably more effective than *A* and *C*, as it was the only tool capable of detecting the bugs in 10.1% of all PUTs. On the other hand, every tool was somewhat effective, and all three of them detected 11.6% of the bugs.

**Fig. 3:**
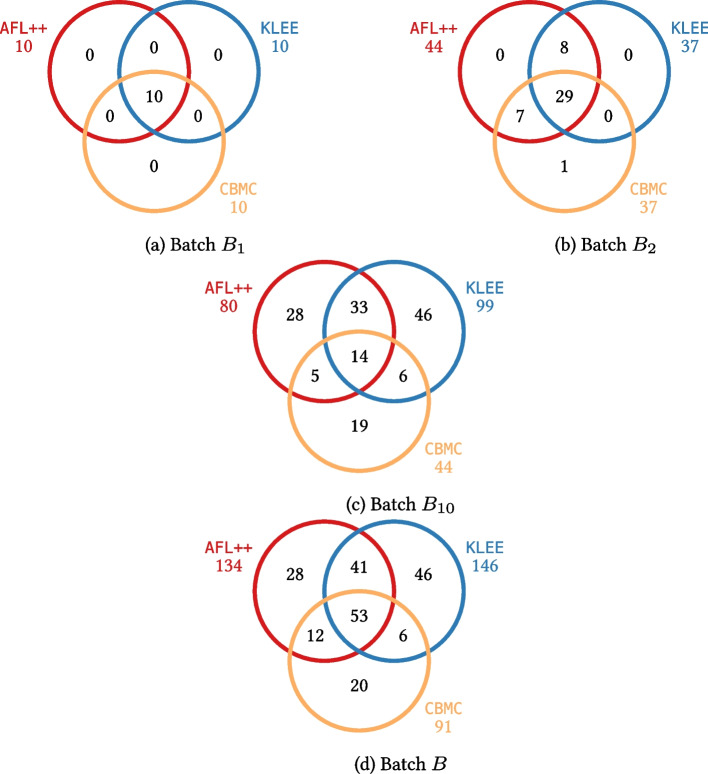
For each batch of PUTs $B_1$, $B_2$, $B_{10}$ and *B* used in the experiments, the Venn diagram reports the number of PUTs in the batch whose bugs were triggered by each tool. The numbers in black in each intersection are the bugs triggered *exclusively* by the corresponding tool combination; the numbers in color below each tool name are the bugs triggered *overall* by the corresponding tool

Among the individual batches of PUTs, $B_{10}$ is the “fairest”, in that it includes PUTs that are challenging for each individual testing tool. In contrast, the PUTs in batches $B_1$ and $B_2$ are generally simple to analyze for most of the tools; and the PUTs in batches $B_{100}$ and $B_{1000}$ are overly complex, so much that no testing tool could detect their bugs in the allotted time. These results are a consequence of the different parameters chosen to create the PUTs in these batches. Overall, these results (also summarized in Fig. 3) suggest that HyperPUT can generate PUTs with bugs that are *fair*, as they are a mix of elusive (highly challenging) bugs and simpler bugs that most practical testing frameworks can discover.

### RQ2: Reproducibility

As expected, all PUTs produced by HyperPUT for our experiments passed the reproducibility checks discussed in Section 4.5. Namely:  

*i)*: Running each PUT on HyperPUT’s generated input triggers the unique bug in the PUT.
*ii)*: The PUTs compile without errors or warnings.
*iii)*: The PUTs behave in the same way regardless of which compiler is used to compile them.
*iv)*: LLVM’s Undefined Behavior Sanitizer does not report any source of undefined behavior in the PUTs.

  These checks confirm that HyperPUT produces PUTs with reproducible seeded bugs, since they are well-formed and behave consistently as expected.

**Fig. 4:**
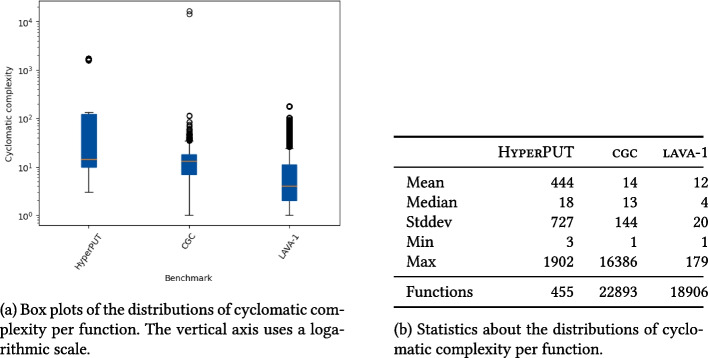
Distributions of cyclomatic complexity per function in three collections of buggy programs: the PUTs in batch *B* generated by HyperPUT, and benchmarks CGC (DARPA CGC 2018) and LAVA-1 (Dolan-Gavitt et al. 2016)

**Fig. 5:**
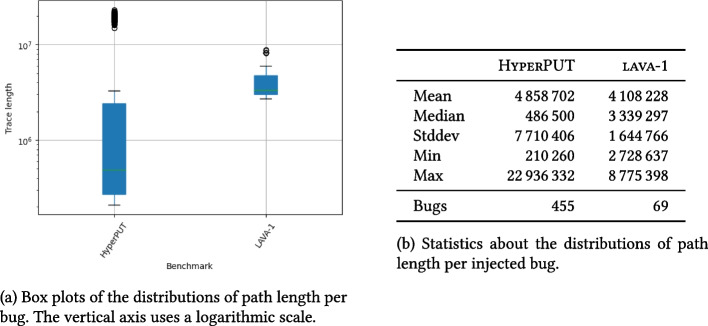
Distributions of the length of the execution path on a bug-triggering input in two collections of buggy programs: the PUTs in batch *B* generated by HyperPUT, and benchmark LAVA-1 (Dolan-Gavitt et al. 2016)

### RQ3: Depth and Rarity

#### Depth

Figure 4 summarizes the distribution of *cyclomatic complexity* measures for the functions in the PUTs generated by HyperPUT (batch *B*), and compares it to the functions featuring in the benchmarks CGC and LAVA-1. HyperPUT can generate very complex PUTs according to this metric: even though some of CGC’s programs are an order of magnitude more complex, HyperPUT’s PUTs cover a broad range of cyclomatic complexities, and are those with the highest average complexity. This is a consequence of the way we configured HyperPUT to generate also large and complex PUTs in batches $B_{100}$ and $B_{1000}$ (as described in Section 4.2). In particular, the outliers in HyperPUT’s box plot in Fig. 4a correspond to several PUTs in batch $B_{1000}$ with very high cyclomatic complexity—hence, among those with the most complex and deep control flow.

Overall, these results suggest that HyperPUT is capable of generating simple as well as complex PUTs, and hence can generate a diverse collection of synthetic buggy programs.

Cyclomatic complexity measures the branching complexity of programs, which is only a proxy for the complexity of the *bugs* that appear in the programs. In principle, a very complex program may have very shallow bugs if they occur in the first few lines of executable code. Path length—the number of instructions executed from program entry until the bug is triggered—better assesses the depth of the synthetic bugs in HyperPUT’s generated PUTs. Figure 5 summarizes the distribution of *path length* for each bug in the PUTs generated by HyperPUT (batch *B*), and compares it to the path length of synthetic bugs in the benchmark LAVA-1.

HyperPUT’s synthetic bugs are deeper on average (mean), but LAVA-1’s bugs are not that far behind, and have a much higher median. In fact, HyperPUT’s have a higher standard deviation, as the batch *B* includes both small PUTs with shallow short-path bugs and large PUTs with bugs that are deeply nested.

As for other measures, this variety is a direct consequence of the way we configured HyperPUT (as described in Section 4.2). Overall, HyperPUT can generate shallow as well as deep bugs, including several that exhibit metrics similar to those of organic bugs.

**Table 7:** Number of KLEE-generated inputs as a measure of bug rarity

(a) Statistics about the number *f* of all test inputs generated by KLEE per bug before triggering the bug in: HyperPUT’s batch $B_{\ge 6}$, manually seeded bugs in TCAS, and synthetic bugs seeded with Apocalypse; the latter two are after (Roy et al. 2018, Fig. 5)			
		(Roy et al. 2018, Fig. 5)	
	HyperPUT	tcas	Apocalypse
Mean	44345	23	345
Median	23244	17	165
Stddev	82429	22	569
Min	29	8	7
Max	486428	152	4366
Bugs	72	41	82
(b) Statistics about the number *t* of bug-triggering test inputs per bug generated by KLEE: HyperPUT’s batch $B_{\ge 6}$, manually seeded bugs in TCAS, and synthetic bugs seeded with Apocalypse; the latter two are after (Roy et al. 2018, Table 4)			
		(Roy et al. 2018, Table 4)	
	HyperPUT	tcas	Apocalypse
Mean	2127 (86)	363	13
Median	1 (1)	213	1
Stddev	6683 (304)	431	51
Min	1 (1)	24	1
Max	40708 (1402)	1805	341
Bugs	72	41	82

#### Rarity

Table 7 shows statistics about the rarity of bugs in HyperPUT’s PUTs in $B_{\ge 6}$, and compares them to the analogous measures reported in Roy et al. (2018, Table 4 and Fig. 5) and in Roy et al. (2018, Table 4) about: *i*) bugs in the TCAS benchmark, which consist of manually seeded bugs in several variants of an organic program; *ii*) bugs seeded using the Apocalypse system (introduced in Roy et al. (2018)) in the same programs of the TCAS benchmark.

According to Table 7a, compared to its behavior on the other benchmarks TCAS and Apocalypse, KLEE needs to generate a much higher number of test inputs before it can detect a bug in HyperPUT’s batch $B_{\ge 6}$. On the other hand, Table 7b suggests that, once KLEE finds the first bug-triggering input, it can fairly easily find *other* bug-triggering inputs in batch $B_{\ge 6}$ if they exist; whereas, on average, KLEE finds one-two orders of magnitude fewer bug-triggering inputs on the other benchmarks. To explain this discrepancy, consider the semantics of transformations FL, PC, and CC, which admit several different reaching inputs (see Section 3.2.1). Once KLEE finds a set of constraints that characterize a triggering input, it can easily find *other* solutions to the same constraints that reach the same error location. For example, transformation PC requires that the input is a palindrome string of length *n*; given one such string, we can get other palindromes by changing any pair of characters at opposite positions in the string. Consequently, the values in parenthesis refer to the number of triggering test cases for PUTs in Batch $B_{\ge 6}$ with non-negligible parameter size ($n>3$ for transformation *PC* and $e>=90$ for transformation FL).

As with other research questions, we demonstrated one way of configuring HyperPUT so that it produces artificial bugs with *some* characteristics comparable to those of other benchmarks of bugs. Users with different requirements could adapt the generation of PUTs to match their needs; for example, one could only include transformations that determine PUTs with a single triggering input (such as a combination of multiple SC with different parameters).

### RQ4: Capabilities

The previous research questions demonstrated that HyperPUT is capable of producing PUTs with bugs with a broad range of characteristics, some comparable to those present in commonly used benchmarks for bug-finding tools. In particular, Section 5.1 suggests that different PUTs are more or less challenging for different bug-finding tools.

**Fig. 6:**
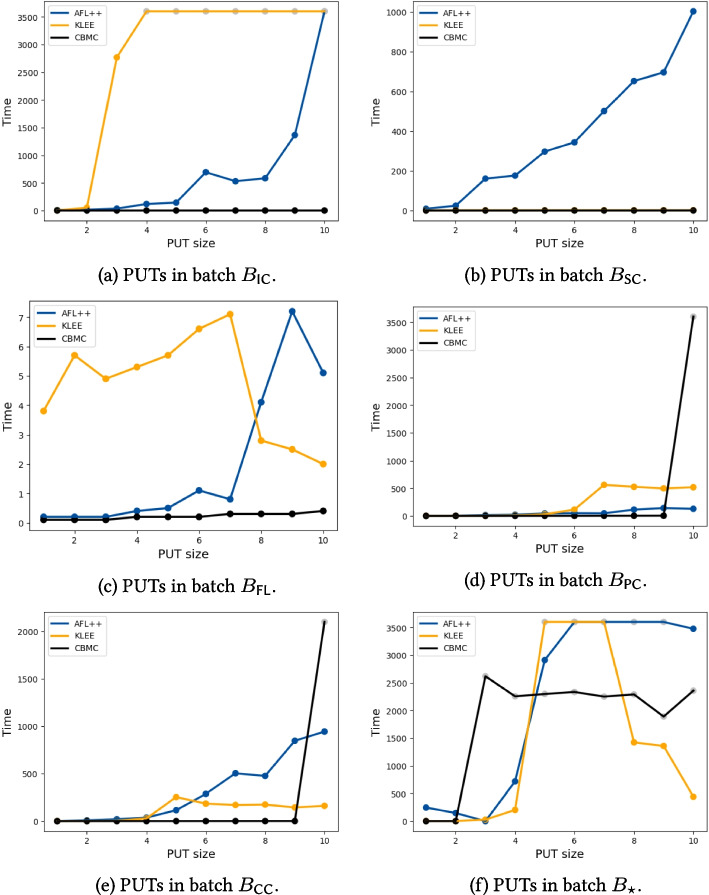
Running time to discover the bug in each PUT in batches $B_{\textsf {IC}}, B_{\textsf {SC}}, B_{\textsf {FL}}, B_{\textsf {PC}}, B_{\textsf {CC}}, B_{\star }$. The horizontal axis enumerates the 10 PUTs in each batch in order of size (number of transformations). The vertical axis measures the running time (in seconds) until the tool terminates or times out (as in all other experiments, we report the average of 4 repeated runs). A colored filled disc indicates that the tool terminated successfully (it discovered the bug); a grayed out circle indicates that the tool terminated or timed out without discovering the bug. Data about AFL++ are in color blue, about CBMC are in color black, about KLEE are in color yellow

To this end, we generated new batches of PUTs $B_{\textsf {IC}}, B_{\textsf {SC}}, B_{\textsf {FL}}, B_{\textsf {PC}}, B_{\textsf {CC}}, B_{\star }$. As described in Section 4.7, PUTs in each batch $B_T$ only use the same transformation *T*, and differ only in their size—measured as the number of repetitions of *T*. This way, we can understand how the characteristics of each transformation challenge a tool’s bug-finding capabilities. Figure 6 plots the running time of the considered testing frameworks when searching for bugs in these PUTs. Unsurprisingly, the performance of a tool clearly depends on the transformations that make up a PUT. Let’s look into each tool’s performance on the different batches. Table 8 provides another qualitative summary of Fig. 6’s experimental results.

**Fig. 7:**
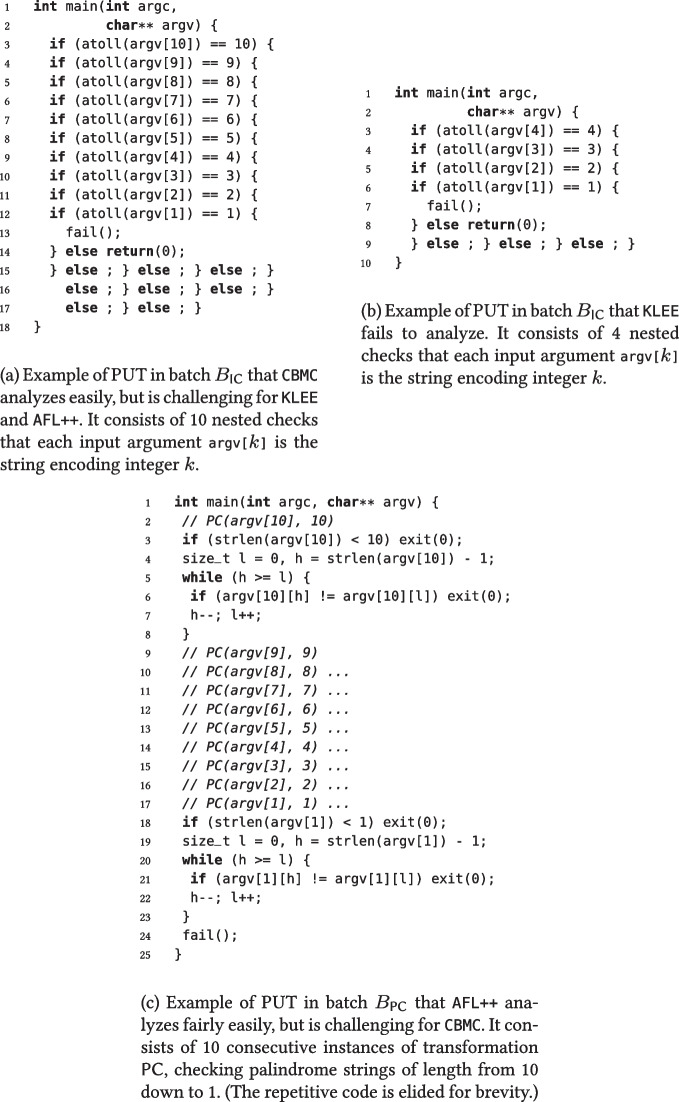
Examples of PUTs that are challenging for different testing frameworks

CBMC is very effective on PUTs using transformations IC, SC, and FL, where it scales effortlessly. PUTs using transformations IC and SC have no loops, and hence CBMC can easily build an exhaustive finite-state abstraction. For example, CBMC found the bug in Fig. 7a’s PUT in less than a second.

**Table 8:** A qualitative summary of which transformations are harder/easier for each testing framework

	transformation				
tool	IC	SC	FL	PC	CC
AFL++	M	M	L	L	L
CBMC	L	L	L	M	M
KLEE	S	L	L	L	L

For each combination of tool/transformation, the table reports the size (among Small, Medium, and Large) of PUTs consisting of repetitions of that transformation that the tool successfully analyzed in Fig. 6’s experiments

PUTs using transformations FL do have loops, but in this case CBMC manages to find a suitable loop unrolling bound that makes the analysis exhaustive without blowing up the search space. In contrast, CBMC’s performance quickly degrades for the largest PUTs using 10 transformations CC and PC (such as the one shown in Fig. 7c); in these case, loops whose exit condition depends on an input string become hard to summarize with a fixed, small unrolling bound past a certain size. Similarly, CBMC’s performance on batch $B_\star$ depends on how many and which transformations are used; in particular, as soon as the randomly generated PUTs include several nested loops with transformations PC or CC, CBMC runs out of resources and terminates in about 40 minutes without detecting the bugs.

KLEE is as effective as CBMC on PUTs using transformation SC. It outperforms CBMC on PUTs using transformations PC and CC, where it scales graciously to the largest PUTs thanks to its symbolic reasoning capabilities. On PUTs using transformation FL, KLEE is always effective, but its running times fluctuate somewhat unpredictably—albeit remaining reasonably low in absolute value. This is probably a result of running KLEE with randomized search (see Table 3), a feature that can speed up the search for bugs but also introduces random fluctuations from run to run. In contrast, KLEE struggles to scale on PUTs using transformation IC both in batch $B_{\textsf {IC}}$ and in batch $B_\star$—such as the one in Fig. 7a, but even with smaller PUTs consisting of just four nested transformations, such as the one in Fig. 7b.

The problem here is not the transformation per se, but rather how it is instantiated in the PUTs generated for the experiments. As we explain in Section 4.2, parameter $v_1$ in transformation IC is instantiated with  , which interprets a string command-line argument as an integer; since KLEE does not have access to the source code of library function  , it treats it as a black box, and hence its constraint solving capabilities are of little use to find efficiently a suitable string argument that  converts to the integer $v_2$ (the transformation’s second parameter, instantiated with a random integer). This also explains the difference in performance with transformation SC, where there is no black-box function involved, and hence KLEE can easily find a suitable input string from the transformation’s condition itself.

AFL++ remains reasonably effective largely independent of which transformations are used; however, its running time tends to grow with the size of the analyzed PUT. This behavior—complementary to KLEE’s and CBMC’s—is a result of AFL++ being a gray box tool. In a nutshell, this means that AFL++ does not have direct access to the source code of the analyzed functions; thus, it cannot extract path constraints from it but has to “guess” them indirectly by trial and error. AFL++’s gray-box strategy, combined with its many heuristics and optimizations, achieves a different trade off than white-box tools like KLEE and CBMC: AFL++ is an overall more flexible tool (in that it is less dependent on the characteristics of the analyzed software), but usually requires more time and has more random fluctuations in its behavior. Another difference is in scalability: AFL++’s analysis time necessarily grows with the size of the inputs; in contrast, symbolic techniques like KLEE are much more insensitive to input size, as long as the complexity of the symbolic constraints does not vary.

Figure 7c depicts an example showcasing AFL++’s effectiveness. Thanks to its speed generating thousands of inputs per second, and to its coverage-driven search that leads to incrementally constructing one suitable input at a time, AFL++ finds a bug-triggering input in about one minute; in contrast, CBMC times out trying to find suitable unrolling factors for the ten loops. In this example, AFL++’s coverage-based heuristics even outperform KLEE, which manages to discover the bug in Fig. 7c but takes about five times longer than AFL++. Still, AFL++’s heuristics and speed run out of steam as the size of the program to be analyzed, and the resulting constraints on failing inputs, increas; therefore, AFL++ times out analyzing the bug in Fig. 7a—which is just a bigger, more complex version of the one in Fig. 7c.

Overall, these results demonstrate how HyperPUT can be used to generate PUTs with heterogeneous characteristics and sizes, which challenge different capabilities of diverse bug-finding techniques.

### Limitations and Threats to Validity

We discuss the main limitations of HyperPUT’s technique, its current implementation, and other threats to the validity of the experiments described in this section, as well as how we mitigated them.

*Construct validity* depends on whether the measurements taken in the experiments reflect the features that are being evaluated. In our experiments, we mainly collected standard measures, such as running time, whether a bug-finding tool managed to trigger a bug, and static (cyclomatic complexity) and dynamic (path length) measures of complexity. For the experiments to answer RQ3, we also counted the number of triggering test cases and generated test cases for each bug—the same measures used by Roy et al. (2018) to assess bug rarity. Using standard measures reduces the risk of threats to construct validity, and helps ensure that our results are meaningfully comparable with those in related work.

Our experiments to answer RQ4 were limited by the transformations currently supported by HyperPUT, and by how we combined them. These restrictions are still consistent with RQ4’s aim, which is to explore HyperPUT’s capabilities to exercise different testing techniques with PUTs of different characteristics.

*Internal validity* depends on whether the experiments adequately control for possible confounding factors. One obvious threat follows from possible bugs in our implementation of HyperPUT. As usual, we mitigated this threat with standard software development practices, such as (manual) regression testing, code reviews, and periodic revisions and refactoring.

To account for fluctuations due to the nondeterministic/randomized behavior of some testing tools, we followed standard practices by repeating each experiment multiple times, and reporting the average values (see Section 4.3). We usually observed only a limited variance in the experiments, which indicates that the practical impact of randomness was usually limited.

Our experiments ran with a timeout of one hour per analyzed bug; it is possible that some experiments would have resulted in success if they had been allowed a longer running time. We chose this timeout as it is standard in such experiments (Roy et al. 2018), and compatible with running a good number of meaningful experiments in a reasonable time. Our experiments showed a considerable variety of behavior, which suggests that the testing tools we used can be successful within this timeout.

**Table 9:** For each research question rq with a certain target property to investigate, the batches of PUTs generated by HyperPUT used in the experimental evaluation of that question, and the sizes (in number of transformations) of those PUTs

rq	Target	Batches	Size
1	fairness	$B = B_1 \cup B_2 \cup B_{10} \cup B_{100} \cup B_{1000}$	1, 2–10, 100, 1000
2	reproducibility	$B = B_1 \cup B_2 \cup B_{10} \cup B_{100} \cup B_{1000}$	1, 2–10, 100, 1000
3	depth	$B = B_1 \cup B_2 \cup B_{10} \cup B_{100} \cup B_{1000}$	1, 2–10, 100, 1000
3	rarity	$B_{\ge 6} \subset B_{10}$	6–10
4	capabilities	$B_{\textsf {IC}} \cup B_{\textsf {SC}} \cup B_{\textsf {FL}} \cup B_{\textsf {PC}} \cup B_{\textsf {CC}} \cup B_{\star }$	1–10

A related threat is in how we configured the testing tools (see Table 3). AFL++, CBMC, and KLEE are highly-configurable tools, and their performance can vary greatly depending on which options are selected. Our goal was not an exhaustive exploration of all capabilities of these tools, but rather a demonstration of their “average” behavior. Correspondingly, we mitigated this threat by: *i*)running each tool with two configurations; *ii*) including the default configuration (with no overriding of default options); *iii*) using common, widely used options.

To answer RQ3 in Section 4.6.2, we compared some measures taken on PUTs generated by HyperPUT with the same measures reported by Roy et al. (2018). Since we did not repeat (Roy et al. 2018)’s experiments in the same environment where we ran HyperPUT, we cannot make strong, quantitative claims about the results of this comparison. This limitation does not, however, significantly threaten our overall answer to RQ3, which is that HyperPUT can generate bugs whose rarity is realistic. Roy et al. (2018)’s experiments are used as a reference for what “realistic” means, whereas our work’s aims are largely complementary.

*External validity* depends on whether the experimental results generalize, and to what extent.

HyperPUT currently generates PUTs with a trivial modular structure, consisting of a single function that only uses a handful of standard C libraries. On the other hand, each function can be structurally quite intricate, with bugs nested deep in the function’s control-flow structure. This is partly a limitation of the current implementation, but also an attempt to focus on generating PUTs that are *complementary* to organic bug-seeded programs. Detecting “deep” bugs is a relevant open challenge in test automation (Böhme et al. 2021), and synthetic buggy programs may be interesting subjects to demonstrate progress in addressing the challenge.

HyperPUT generates programs in C since this is a widely popular target for the research on automated testing and fuzzing. The ideas behind HyperPUT can certainly be applied to other programming languages, possibly with different results.

Similarly, the choice of transformations currently supported by HyperPUT obviously limits its broader applicability. HyperPUT’s implementation is extensible with new transformations; deciding which ones to add depends on the goal of the experiments one would like to make.

## Discussion

The bulk of this paper described HyperPUT’s experimental evaluation, which aimed at demonstrating how HyperPUT can be flexibly configured to produce diverse PUTs with various characteristics. As summarized by Table 9, we customized HyperPUT’s parameters so as to produce PUTs that are suitable to address each research question.

For research questions RQ1 and RQ2, we produced a wide collection of PUTs (batch *B*), ranging from trivial ones with a single transformation (batch $B_1$) up to very large and complex ones with up to 1000 transformations (batch $B_{1000}$). Each PUT randomly combines any of the five transformations currently supported by HyperPUT (see Section 3.1). At least one among the testing frameworks AFL++, CBMC, and KLEE detected 45.3% of all bugs in batch *B*, 58.0% of all bugs in batches $B_1, B_2, B_{10}, B_{100}$ and 80.8% of all bugs in batches $B_1, B_2, B_{10}$. This demonstrates that HyperPUT can produce PUTs that are *fair*, that is, neither trivial nor completely inaccessible for the capabilities of the state-of-the-art testing frameworks. All PUTs produced in the experiments were configured so that HyperPUT can also produce a triggering input that reaches the location of the seeded bugs. We confirmed that the triggering inputs produced by HyperPUT work in all experiments, as well as that the PUTs compile correctly without warnings (thus answering RQ2).

The complex PUTs in batches $B_{100}$ and $B_{1000}$ are also useful to demonstrate that HyperPUT can easily produce *deep* bugs (research question RQ3), nested within complex and long control flow paths. Investigating *rarity* turned out to be more subtle. On the one hand, we cannot assess rarity using very large, overly complex PUTs: if no testing framework can generate a triggering input for a bug within a reasonable time, any measure of rarity would be undefined. On the other hand, including very small, trivial PUTs in the experiments on rarity would also be pointless, as we do not expect these PUTs to challenge in any meaningful way the capabilities of state-of-the-art testing frameworks. In our experiments, we selected a batch $B_{\ge 6}$ of “Goldilocks” PUTs that are neither too complex nor too trivial, which we used to demonstrate how HyperPUT compares to other benchmarks in terms of rarity. As remarked in Section 5.3.2, one could configure HyperPUT differently to produce bugs that are more rare (for example, using only transformations that determine bugs with unique triggering inputs)—in exchange for losing some heterogeneity.

With research question RQ4, we wanted to discriminate between the *capabilities* of different testing frameworks, connecting them to different features of the PUTs we supply to them. To this end, we generated different batches of PUTs that consist of repetitions of a *single* transformations among those of Table 2. These experiments confirmed how the different testing techniques of fuzzing (AFL++), model checking (CBMC), and symbolic execution (KLEE) are challenged by different program features: CBMC could not complete the analysis of PUTs with loops that cannot be summarized symbolically; KLEE struggled especially with complex path constraints (for example in deeply nested conditionals); AFL++’s heuristics make it quite flexible, but tend to fail when analyzing bugs with few triggering inputs.

Based on the above discussion, here are some high-level *guidelines* that users of HyperPUT can follow to generate PUTs that meet their requirements. Specifically, requirements refer to different *characteristics* of the generated PUTs that one wants to enforce:  

**Size**: The *number of transformations* is the fundamental determinant of the size of the generated PUTs. Each transformation corresponds to a parametric snippet of code; hence, each transformation instance adds as many lines of code as in the snippet.
**Nesting**: **depth** HyperPUT generates PUTs given *transformation sequences* (Section 3.2), which chain transformations in different ways. This gives user control over the nesting depth of the resulting PUTs, as measured by metrics such as cyclomatic complexity.
**Triggering**: **inputs** As we discussed in Section 3.2.1, HyperPUT can produce triggering inputs with every bug it generates provided we compose transformations under certain restrictions. This feature of HyperPUT can also be extended, for example to produce multiple triggering inputs, or to support the generation of triggering inputs with new user-defined transformations.
**Bug**: **kinds** While all our experiments used simple bugs consisting of assertion failures, HyperPUT can use an arbitrary piece of code as seeded bug. Users could take advantage of this capability in order to produce PUTs that exercise *specialized* program analysis frameworks that can only detect certain kinds of bugs (e.g., null-pointer dereferences Banerjee et al. 2019).
**Program**: **features** To generate PUTs with specific, homogeneous features (for example, that do not include loops, or that only use variables of type string), users of HyperPUT select some or all of the *transformation categories* to be used for generation. The current version of HyperPUT is limited to a core subset of features of the C programming language, which was sufficient to demonstrate its capabilities in our experiments. However, one can still define new transformations, and combine them with the others in HyperPUT, so as to cater to their specific needs.

## Conclusions

In this paper, we presented HyperPUT, a technique and tool to generate PUTs (Program Under Tests) with seeded bugs automatically, according to desired characteristics. The PUTs generated by HyperPUT can be useful as experimental subjects to assess the capabilities of bug-finding tools, and how they change according to the characteristics of the analyzed PUT. To demonstrate this, we generated hundreds of PUTs using HyperPUT, and ran the popular bug-finding tools AFL++, CBMC, and KLEE on them. Our experiments suggest that HyperPUT can generate heterogeneous collections of PUTs, with several characteristics that resemble those of “ecologically valid” bugs (Roy et al. 2018).

The implementation of HyperPUT is extensible, so that users can easily add transformations and parameters to configure the generation of bugs according to the intended usage. As future work, we plan to further extend the flexibility of HyperPUT, so that it can also automate the process of analyzing its experimental results (without the manual intervention normally needed to study the produced data or to generate graphical representations from it), or so that it can extend an existing program with new functions and seeded bugs.

## Data Availability

The prototype implementation of HyperPUT is available in a public repository (HyperPUT 2022).

## Footnotes

1 To corroborate the hypothesis that including more PUTs of small size would not significantly affect the experimental results about fairness, we ran a few additional experiments. First, we added 43 new PUTs of “small” sizes 2, 3, and 4 to batch $B_{10}$, so that the size distribution of this extended batch $B_{10}^+$ is more balanced. We found that 90.7% of the added bugs were detected by at least one tool among AFL++, CBMC, and KLEE; this percentage is considerably higher than the 75.5% obtained with the original batch $B_{10}$ (see Table 6). Correspondingly, the overall percentage over the extended batch $B_{10}^+$ only grows by 2.7% (from 75.5% to 78.2%). This confirms that “small” PUTs are not particularly discriminating, and validates our selection of batch $B_{10}$.

2 Extending HyperPUT to support different kinds of bugs (for example those targeted by termination checkers (Cook et al. 2011)) is an interesting direction for future work.

3 Measured using CCCC (Littlefair 2005) and PMCCABE (Bame 2023) open source tools.

4 This metric has been measured using the profiling tool Cachegrind (Nethercote 2004).

5 Corresponding to batch $B_{1} \cup B_{2} \cup B_{10}$.
